# Inflammatory changes in the airways of mice caused by cigarette smoke exposure are only partially reversed after smoking cessation

**DOI:** 10.1186/1465-9921-11-99

**Published:** 2010-07-22

**Authors:** Saskia Braber, Paul AJ Henricks, Frans P Nijkamp, Aletta D Kraneveld, Gert Folkerts

**Affiliations:** 1 Division of Pharmacology, Utrecht Institute for Pharmaceutical Sciences, Faculty of Science, Utrecht University, Utrecht, The Netherlands

## Abstract

**Background:**

Tobacco smoking irritates and damages the respiratory tract and contributes to a higher risk of developing lung emphysema. At present, smoking cessation is the only effective treatment for reducing the progression of lung emphysema, however, there is hardly anything known about the effects of smoking cessation on cytokine and chemokine levels in the airways. To the best of our knowledge, this is the first reported *in vivo *study in which cytokine profiles were determined after cessation of cigarette smoke exposure.

**Methods:**

The severity of airway remodeling and inflammation was studied by analyzing alveolar enlargement, heart hypertrophy, inflammatory cells in the bronchoalveolar lavage fluid (BALF) and lung tissue and by determining the cytokine and chemokine profiles in the BALF of A/J mice exposed to cigarette smoke for 20 weeks and 8 weeks after smoking cessation.

**Results:**

The alveolar enlargement and right ventricle heart hypertrophy found in smoke-exposed mice remained unchanged after smoking cessation. Although the neutrophilic inflammation in the BALF of cigarette smoke-exposed animals was reduced after smoking cessation, a sustained inflammation in the lung tissue was observed. The elevated cytokine (IL-1α and TNF-α) and chemokine (CCL2 and CCL3) levels in the BALF of smoke-exposed mice returned to basal levels after smoking cessation, while the increased IL-12 levels did not return to its basal level. The cigarette smoke-enhanced VEGF levels did not significantly change after smoking cessation. Moreover, IL-10 levels were reduced in the BALF of smoke-exposed mice and these levels were still significantly decreased after smoking cessation compared to the control animals.

**Conclusion:**

The inflammatory changes in the airways caused by cigarette smoke exposure were only partially reversed after smoking cessation. Although smoking cessation should be the first step in reducing the progression of lung emphysema, additional medication could be provided to tackle the sustained airway inflammation.

## Introduction

There are currently more than 1.3 billion tobacco smokers worldwide according to the World Health Organization (WHO) [1]. Cigarette smoke contains more than 4000 hazardous chemical compounds, of which 200 are highly toxic [2]. It is generally accepted that cigarette smoking is the most important risk factor for the development and progression of chronic obstructive pulmonary disease (COPD) and accounts for about 80% of COPD cases [3,4]. COPD, a term referring to two lung diseases: chronic bronchitis and emphysema, is characterized by an airflow limitation that is not fully reversible. The airflow limitation is usually both progressive and associated with an abnormal inflammatory response of the lungs to noxious particles or gases [5]. Pulmonary hypertension and right ventricular failure are also often associated with COPD [6,7]. Since a chronic airway inflammation with alveolar wall destruction and airway remodeling is central to the pathogenesis of COPD, it is not surprising that several types of inflammatory cells play a role in this condition [8]. Increased numbers of macrophages and neutrophils are observed in sputum and bronchoalveolar lavage fluid (BALF) of COPD patients [9-11]. In addition, COPD patients have elevated levels of T-lymphocytes, in particular CD8+ cells, in lung parenchyma and airways [11-14]. Migration and activation of inflammatory cells to the lung is regulated by the release of different mediators, including proteases, cytokines and chemokines secreted by a variety of inflammatory and resident cells. These mediators contribute to the chronic inflammatory process with tissue damage and repair processes seen in emphysema [15,16]. Several cytokines and chemokines have been implicated in the airway inflammation in COPD. Increased levels of interleukin-8 (IL-8), interleukin-12 (IL-12), tumour-necrosis factor-α (TNF-α), monocyte chemotactic protein-1 (MCP-1; CCL-2), and macrophage inflammatory protein-1α (MIP-1α; CCL3) have been observed in COPD patients [9,17-21]. In general, the treatments available for COPD reduce the number and severity of exacerbations and relieve symptoms, but do not tackle the cause of the disease and have a limited effect on slowing down the progression of lung damage [22]. At present, smoking cessation is the only effective treatment for avoiding or reducing the progression of COPD [23]. However, there is contradictory evidence regarding the effect of smoking cessation on airway inflammation associated with COPD. Several studies in COPD patients reported that smoking cessation improves respiratory symptoms, reduces loss of pulmonary function and decreases lung inflammation [24-28], while other studies have shown that smoking cessation fails to reverse the chronic airway inflammation [29-32]. Unfortunately, there is insufficient evidence regarding the effects of smoking cessation on cytokine and chemokine levels, which do play an important role in airway inflammation and tissue remodeling seen in COPD. Therefore, a murine model of cigarette smoke-induced lung emphysema was used to investigate the effect of smoking cessation on airway remodeling and pulmonary inflammation. The severity of airway remodeling and inflammation was studied by determining alveolar enlargement, heart hypertrophy, inflammatory cells in the bronchoalveolar lavage fluid (BALF) and lung tissue and by analyzing the cytokine and chemokine profiles in the BALF of mice exposed to cigarette smoke for 20 weeks and 8 weeks after smoking cessation.

## Materials and methods

### Animals

Female A/J mice, 9-14 weeks old (Charles River Laboratories) were housed under controlled conditions in standard laboratory cages. They were provided free access to water and food. All *in vivo *experimental protocols were approved by the local Ethics Committee and were performed under strict governmental and international guidelines on animal experimentation.

### Cigarette smoke exposure

Female A/J mice were divided into three groups. The first group was exposed to room air for 20 weeks, the second group was exposed to cigarette smoke for 20 weeks and the third group was exposed to cigarette smoke for 20 weeks followed by a period of 8 weeks without cigarette smoke exposure. 20-weeks-old mice are adult mice and should have almost no alveolar growth in the additional 8 weeks [33,34]. In the life-span of a laboratory mouse 20 weeks smoking and 8 weeks smoking cessation represents approximately 21 years smoking and 8 years smoking cessation in humans. The mice were exposed in whole-body chambers to air (sham) or to diluted mainstream cigarette smoke from the reference cigarettes 2R4F (University of Kentucky, Lexington, Kentucky) using a smoking apparatus. Exposures were conducted 4 h/day (with a 30/60-minute fresh air break after each hour of exposure), 5 days/week for 20 weeks to a target cigarette smoke concentration of 750 μg total particulate matter/l (TPM/l). This TPM concentration was reached after an adaptation period of 1 week, starting with a TPM concentration of 125 μg TPM/l. The mass concentration of cigarette smoke TPM was determined by gravimetric analysis of Cambridge filter samples. The carbon monoxide (CO) was monitored continuously and was around 800 ppm. The nicotine concentration in the smoke was approximately 40 μg/l. The sample sites were located in the middle of the exposure chamber at the breathing zone. The mice were sacrificed 16-24 hours after the last air or smoke exposure, or after the smoke-free period of 8 weeks.

### Histology and morphometric analysis

Mice (n = 4-5), used for morphometric analysis, were sacrificed by an i.p. injection with an overdose of pentobarbital (Nembutal™, Ceva Santé Animale, Naaldwijk, The Netherlands). The lungs were fixated with a 10% formalin infusion through the tracheal cannula at a constant pressure of 25 cm H_2_O. After excision, the volume of the fixed lungs was measured by fluid displacement. Then, the left lung was immersed in fresh fixative for at least 24 h, after which it was embedded in paraffin. After paraffin embedding, 5 μm sections were cut and stained with hematoxylin/eosin (H&E) according to standard methods. These histological lung sections were used to determine lung inflammation and pigmented macrophages. Lung inflammation was scored by a treatment-blind observer. The degree of peribronchial and perivascular inflammation was evaluated on a subjective scale of 0-3, as described elsewhere [35,36]. A value of 0 was assigned when no inflammation was detectable, a value of 1 was adjudged for occasional cuffing with inflammatory cells, a value of 2 when most bronchi or vessels were surrounded by a thin layer (one to five cells thick) of inflammatory cells, and a value of 3 was given when most bronchi or vessels were surrounded by a thick layer (more than five cells thick) of inflammatory cells. Total lung inflammation was defined as the average of the peribronchial and perivascular inflammation scores. Four lung sections per mouse were scored and inflammation scores were expressed as a mean value. Morphometric assessment of emphysema, included determination of the average inter-alveolar distance, was estimated by the mean linear intercept (Lm) analysis. The Lm was determined by light microscopy at a total magnification of 100×, whereby 24 random photomicroscopic images per left lung tissue section were evaluated by microscopic projection onto a reference grid. By dividing total grid length by the number of alveolar wall-grid line intersections, the Lm (in μm) was calculated [37].

### Bronchoalveolar lavage

Immediately after i.p. injection with an overdose of pentobarbital, the lungs of a separate group mice (n = 4-5) were lavaged 4 times through a tracheal cannula with 1 ml saline (NaCl 0.9%), pre-warmed at 37°C. The first lavage was performed with 1 ml saline containing a mixture of protease inhibitors (Complete Mini, Roche Applied Science, Penzberg, Germany). After centrifuging the bronchoalveolar lavage fluid at 4°C (400 g, 5 min), the supernatant of the first ml was used for cytokine analysis and the cell pellets of the 4 lavages were used for cell counts. The 4 cell pellets, kept on ice, were pooled per animal and resuspended in 150 μl cold saline. After staining with Türk solution, total cell counts per lung were made under light microscopy using a Burker-Turk chamber. Differential cell counts were performed on cytospin preparations stained by DiffQuick™(Dade A.G., Düdingen, Switzerland). Cells were identified as macrophages, neutrophils and lymphocytes according to standard morphology. At least 200 cells were counted and the absolute number of each cell type was calculated.

### Right ventricular hypertrophy measurement

The right ventricle was removed from lower heart after removal of the atria. The right ventricle and the left ventricle plus septum were weighed and the ratio of the weights was calculated as follows: (right ventricle)/(left ventricle + septum) [38,39].

### Measurement of cytokines and chemokines

A standard mouse cytokine 20-plex assay was used to determine cytokine and chemokine concentrations in the BALF (n = 4-5) according to the manufacturer's instructions (Luminex; Biosource, Invitrogen, Breda, The Netherlands). The most relevant cytokines and chemokines (IL-1α, IL-10, IL-12, TNF-α, CCL2, CCL3, VEGF and macrophage inflammatory protein-2 (MIP-2; CXCL2)) were discussed in this study. The concentrations of these cytokines and chemokines were expressed as pg/ml BALF.

### Statistical analysis

Experimental results were expressed as mean ± S.E.M. Differences between groups were statistically determined by an unpaired two-tailed Student's *t*-test using GraphPad Prism (Version 4.0). Results were considered statistically significant when P < 0.05.

## Results

### Alveolar enlargement induced by cigarette smoke exposure is irreversible

**Figure 1:**
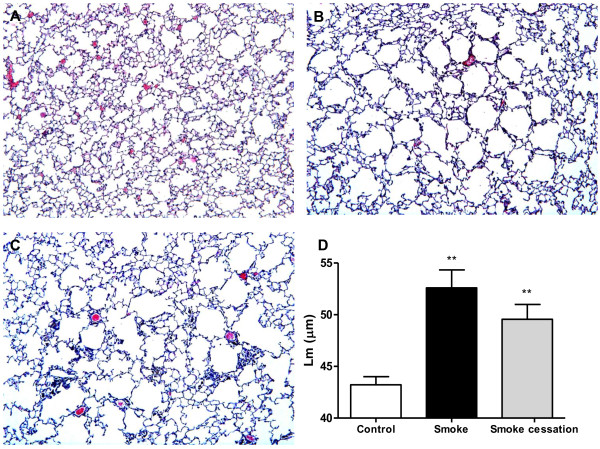
**Cigarette smoke-induced alveolar enlargement is irreversible**. Representative photomicrographs of hematoxylin and eosin stained lung tissue of air-exposed mice (A), smoke-exposed mice (B), smoke-exposed mice 8 weeks after smoking cessation (C). Magnification, ×100. Mean linear intercept (Lm) values of mice exposed to air (white bar), mice exposed to cigarette smoke for 20 weeks (black bar) and mice exposed to cigarette smoke for 20 weeks plus a smoking cessation period of 8 weeks (grey bar) (D). n = 4-5 animals per group. Values are expressed as mean +/- S.E.M. **P ≤ 0.01; significantly different from the control group.

The histological lung sections of the smoke-exposed mice showed an increased air space enlargement and destruction (Fig. 1B) compared with the air-exposed mice (Fig. 1A). The alveolar enlargement is still present after a smoking cessation period of 8 weeks (Fig. 1C). The mean linear intercept, a quantification method for alveolar size, was used to quantify the presence and severity of emphysema [37]. Significant airspace enlargement was observed in mice after 20 weeks exposure to cigarette smoke (Fig. 1D). Furthermore, airspace enlargement induced by cigarette smoke exposure was not reversible, since the increase in Lm was not significantly reduced after a period of 8 weeks without exposure to cigarette smoke (Fig. 1D).

### Right ventricle heart hypertrophy related to cigarette smoke exposure is irreversible

**Figure 2:**
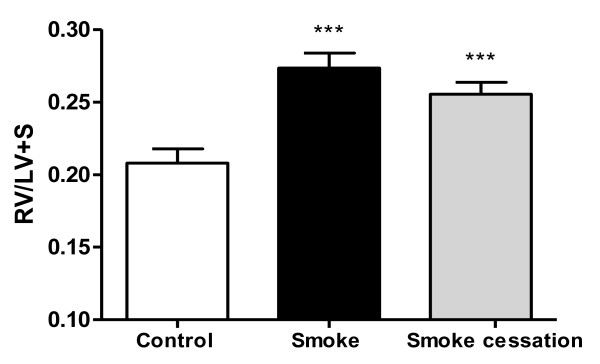
**Cigarette smoke-induced right ventricle heart hypertrophy is irreversible**. Right ventricle (RV) and left ventricle (LV) + septum (S) were dissected after 20 weeks air exposure (white bar), after 20 weeks smoke exposure (black bar) and after 20 weeks smoke exposure plus a smoking cessation period of 8 weeks (grey bar) to determine their weight ratio (RV(LV+S)). n = 6-7 animals per group. Values are expressed as mean +/- S.E.M. ***P ≤ 0.001; significantly different from the control group.

Twenty weeks cigarette smoke exposure caused right ventricular heart hypertrophy (Fig. 2). The right ventricular mass was proportionally greater than the rest of the lower heart (left ventricle and septum) in smoke-exposed mice compared to air-exposed mice. Moreover, right ventricle heart hypertrophy was not reversible after a period of 8 weeks without cigarette smoke exposure, because the heart hypertrophy ratio (RV/LV +S) was not significantly decreased in the smoking cessation group compared to smoke-exposed group.

### Lung volume increase after cigarette smoke exposure is irreversible after smoking cessation

**Figure 3:**
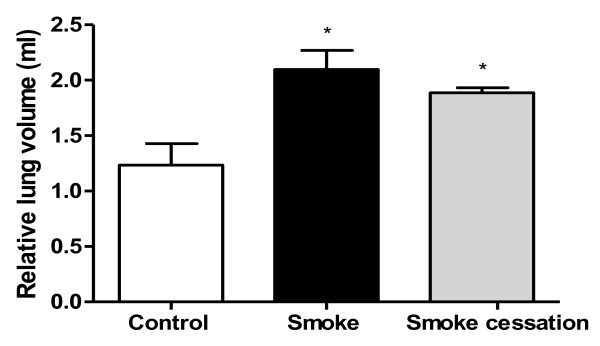
**Lung volume increase after cigarette smoke exposure is not reversible after smoking cessation**. The relative lung volume was measured by fluid displacement. The relative lung volumes were determined after 20 weeks air exposure (white bar), after 20 weeks smoke exposure (black bar) and after 20 weeks smoke exposure plus a smoking cessation period of 8 weeks (grey bar). n = 4-5 animals per group. Values are expressed as mean +/- S.E.M. *P ≤ 0.05; significantly different from the control group.

It has been demonstrated that chronic inflammation in the airways ultimately leads to alveolar enlargement, increased pulmonary compliance as well as enhanced lung volumes [40]. We measured the lung volumes in the murine lung emphysema model and the lung volume was significantly increased in mice exposed to cigarette smoke for 20 weeks compared to the control mice (Fig. 3). After a period of 8 weeks without cigarette smoke exposure, the lung volume was still significantly enhanced compared to the control group.

### Smoking cessation reduces the inflammatory cell influx in bronchoalveolar lavage fluid

Progression of COPD is associated with the accumulation and activation of inflammatory cells in the BALF. In the present lung emhysema model, the total number of inflammatory cells was 5-fold increased in the BALF after 20 weeks of cigarette smoke exposure (Table 1). Differential cell counts demonstrated that most of the cells in the BALF of the air-exposed mice were macrophages, with a few neutrophils and lymphocytes. The number of all these inflammatory cells in the BALF was significantly increased after cigarette smoke exposure, especially the neutrophils. Cigarette smoke exposure also affected the BALF cell composition, since there was a shift observed from mainly macrophages in the control animals towards neutrophils in the BALF of smoke-exposed mice. After smoking cessation of 8 weeks, we found a significant decline in inflammatory cells in the BALF, although the total cell number was still significant different compared to the control group (Table 1). First, the amount of neutrophils was strongly reduced after smoking cessation, but these cell numbers were still significantly increased compared to the control mice. The macrophages were also decreased compared to the smoke-exposed mice, however these numbers were not returned to basal levels. Finally, the cigarette smoke-induced increase of lymphocytes was not changed after cessation of cigarette smoke exposure. These results indicate that smoking cessation leads to a reduction in inflammatory cell types and a change in cell composition in the BALF, mainly caused by a decline in neutrophils.

**Table 1:** Immune cells in BALF recovered from air-exposed mice, smoke-exposed mice and smoke-exposed mice 8 weeks after smoking cessation.

	Control	Smoke	Smoke cessation
Total cell count, × 10^4^	30.0 ± 3.2	140.4 ± 2.6 ***	52.8 ± 5.0 ** ^^^
Differential cell count, × 10^4^			
Macrophages	29.2 ± 3.1	56.1 ± 1.1 ***	42.4 ± 4.2 * ^
Neutrophils	0.27 ± 0.1	79.9 ± 3.5 ***	6.1 ± 0.5 *** ^^^
Lymphocytes	0.51 ± 0.1	4.4 ± 1.0 *	4.4 ± 1.0 *

n = 4-5 animals per group. Values are expressed as mean +/- S.E.M. *P ≤ 0.05, **P ≤ 0.01, ***P ≤ 0.001; significantly different from the control group. ^P ≤ 0.05, ^^^ P ≤ 0.001; significantly different from the smoke group.

### Lung inflammation is still present in lung tissue after smoking cessation

**Figure 4:**
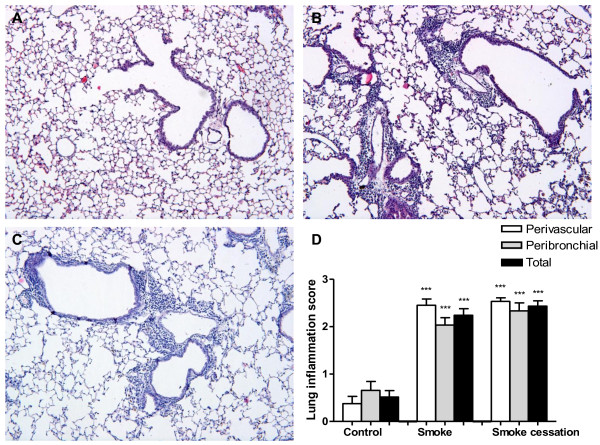
**Lung inflammation is still present in lung tissue after smoking cessation**. Representative photomicrographs of hematoxylin and eosin stained lung tissue of air-exposed mice (A), smoke-exposed mice (B), smoke-exposed mice 8 weeks after smoking cessation (C). Magnification, ×100. The histological sections were scored for the presence of peribronchial and perivascular inflammation (D). Total lung inflammation was defined as the average of the peribronchial and perivascular inflammation scores. n = 4-5 animals per group. Values are expressed as mean +/- S.E.M. ***P ≤ 0.001; significantly different from the control group.

Histological lung sections demonstrated that pulmonary inflammation with peribronchial and perivascular inflammatory cell infiltrates was present in the airways of smoke-exposed mice (Fig. 4B). The air-exposed animals had no detectable lung inflammation (Fig. 4A). The smoking cessation group showed that the peribronchial and perivascular airway inflammation was still present after a smoke-free period of 8 weeks (Fig. 4C), since there was no notable difference in the leukocyte aggregates compared to those found in smoke-exposed lungs. The scores of peribronchial, perivascular and total lung inflammation were significantly increased after 20 weeks cigarette smoke exposure compared to air-exposed mice and these scores were still significantly enhanced after a smoking cessation period of 8 weeks (Fig. 4D).

**Figure 5:**
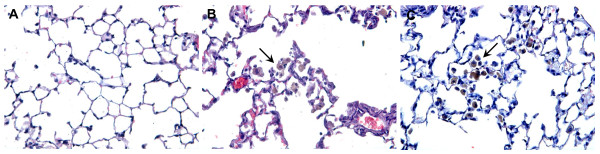
**Pigmented macrophage accumulation in the lung tissue before and after smoking cessation**. Representative photomicrographs of hematoxylin and eosin stained lung tissue of air-exposed mice (A), smoke-exposed mice (B), smoke-exposed mice 8 weeks after smoking cessation (C). n = 4-5 animals per group. Magnification, ×400.

Moreover, there was an accumulation of brown-pigmented macrophages in lung tissue of smoke-exposed mice (Fig. 5B) compared to the lung tissue of the control mice (Fig. 5A). These pigmented macrophages were still present after a smoking cessation period of 8 weeks (Fig. 5C).

### The effect of smoking cessation on smoke-induced changes in cytokine and chemokine levels in BALF

**Figure 6:**
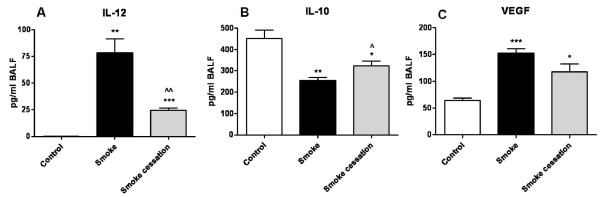
**The effect of smoking cessation on smoke-induced changes in cytokine and chemokine levels in BALF**. Levels of the pro-inflammatory cytokine IL-12 (A), the regulatory cytokine IL-10 (B) and the growth factor VEGF (C) in the BALF of air-exposed mice (white bars), smoke-exposed mice (black bars), smoke-exposed mice 8 weeks after smoking cessation (grey bars). n = 4-5 animals per group. Values are expressed as mean +/- S.E.M. *P ≤ 0.05, **P ≤ 0.01, ***P ≤ 0.001; significantly different from the control group. ^P ≤ 0.05, ^^P ≤ 0.01; significantly different from the smoke group.

The levels of different cytokines and chemokines (IL-1α, IL-10, IL-12, TNF-α, CCL2, CCL3 and VEGF) were measured in the BALF of control mice and in smoke-exposed mice before and after smoking cessation. Differences between the cytokine/chemokine profiles in the BALF before and after smoking cessation were observed. The concentrations of the pro-inflammatory cytokines IL-1α and TNF-α were significantly elevated in the BALF of the cigarette smoke-exposed mice compared to the air-exposed mice (IL-1α: control: 0 pg/ml BALF versus smoke: 73.7 ± 8.7 pg/ml BALF, P < 0.001; TNF-α: control: 17.1 ± 0.3 pg/ml BALF versus smoke: 33.1 ± 2.6 pg/ml BALF, P < 0.01). Both IL-1α and TNF-α returned completely to basal levels after smoking cessation. The cigarette smoke-enhanced IL-12 levels in the BALF did not completely return to its basal level after smoking cessation (Fig. 6A). In contrast to the pro-inflammatory cytokines, the levels of the regulatory cytokine IL-10 were significantly decreased in the BALF after cigarette smoke exposure. Although IL-10 levels were rising after smoking cessation, the smoke-induced reduction was still significantly different from the control group (Fig. 6B). Furthermore, the chemokine levels CCL2 and CCL3 were increased in the BALF of cigarette smoke-exposed mice as compared to the control mice (CCL2: control: 17.8 ± 0.2 pg/ml BALF versus smoke: 298.8 ± 47.7 pg/ml BALF, P < 0.01; CCL3: control: 12.1 ± 3.7 pg/ml BALF versus smoke: 133.6 ± 26.8 pg/ml BALF, P < 0.01), while these chemokines returned completely towards basal levels after smoking cessation. The VEGF levels were enhanced in the BALF after chronic cigarette smoke exposure and were still significantly elevated compared to the air-exposed mice after 8 weeks smoking cessation (Fig.6C).

Since no CXCL2 levels were detected in the BALF of the smoke-exposed mice, CXCL2 levels were also examined in the lung homogenates of these animals. A significant increase of the CXCL2 concentration was observed in the lung homogenates of the smoke-exposed mice (4820.7 ± 820.1 pg/ml/mg protein, P < 0.05) compared to the control animals (1108.1 ± 727.2 pg/ml/mg protein). After smoking cessation the smoke-induced increase of CXCL2 levels was still evident (4175.6 ± 1338.6 pg/ml/mg protein).

## Discussion

This study investigated the effects of smoking cessation on airway remodeling and pulmonary inflammation. First, airspace enlargement in the animal model for lung emphysema was evident after 20 weeks cigarette smoke exposure. This enlargement was not significant reduced after smoking cessation, suggesting that induction of lung emphysema by alveolar wall destruction is not reversible. These findings are in agreement with the *in vivo *data of Wright and Sun [41] and March et al. [42], who demonstrated that emphysema was still present in guinea pigs and mice after smoke exposure followed by a smoking cessation period. Vernooy et al. [43] also found that long-term LPS exposure results in irreversible alveolar enlargement in mice. The effect of cigarette smoke is believed to be strain dependent. A/J mice were used in the present COPD model, since this strain is characterized as moderately susceptible to the development of lung emphysema and to the lung inflammatory response after acute cigarette smoke exposure [44,45]. The persistent emphysema observed in the present murine model is also similar to findings in people who have stopped smoking. The alveolar enlargement and destruction seen in lung emphysema is generally thought to be irreversible [46-48]. Besides the determination of lung emphysema, we were interested in the lung volume. In the current study, cigarette smoke-exposed mice showed a significantly increased relative lung volume compared to the air-exposed mice, which is a characteristic feature of lung emphysema [40]. This lung volume was still significantly enhanced after smoking cessation, which supported the irreversible alveolar changes after cigarette smoke exposure.

Furthermore, right ventricle heart hypertrophy was found in mice exposed to cigarette smoke, indicating changes in the structure of the heart. Other authors also demonstrated right ventricle heart hypertrophy as well in animal models for lung emphysema as in COPD patients [6,7,38,39,49]. A possible explanation for the development of right ventricle heart hypertrophy could be pulmonary hypertension, caused by hypoxic pulmonary vasoconstriction or remodeling of the pulmonary vessels, two important complications of COPD [6,50,51]. VEGF is identified as an endothelial cell specific growth factor that contributes to angiogenesis and vascular permeability [52]. In the current study the increased VEGF levels observed in the BALF of the smoke-exposed mice could be involved in the pulmonary vascular remodeling as a result of pulmonary hypertension, ultimately leading to right ventricle heart hypertrophy. An enhanced expression of VEGF was also observed in the pulmonary vessels and arteries of COPD patients, suggesting an important role for VEGF in the development of pulmonary hypertension [53,54]. However, other studies suggest that VEGF may have a protective role in the development of pulmonary hypertension [55-57]. Like alveolar enlargement, the right ventricle heart hypertrophy and the increased VEGF in the BALF were irreversible after smoking cessation. It is possible that the pulmonary hypertension continued after the recovery period due to the sustained lung damage and elevated VEGF levels, which could lead to the ongoing heart hypertrophy. It remains to be determined whether right ventricle heart hypertrophy is directly related to lung emphysema or whether other factors can play a role in the development and maintaining of heart hypertrophy in COPD patients.

Airway inflammation was present in the airways of mice exposed to cigarette smoke as shown by an increase in total cell number in the BALF and by inflammatory cell infiltration in the lung tissue. Analysis of differential cell counts in BALF revealed a significant increase in the number of macrophages, neutrophils and lymphocytes in the smoke-exposed mice compared to air-exposed mice, which is described in several *in vivo *studies [58-61]. The histological lung sections and lung inflammation scores of the smoke-exposed mice confirmed pulmonary inflammation with perivascular and peribronchial cellular infiltrates, which has also been demonstrated in other *in vivo *studies [62,63]. After smoking cessation, the reduced numbers of inflammatory cells in the BALF did not correlate with the sustained inflammatory cell infiltration observed in lung tissue. These results support the studies by Seagrave et al. [64] and March et al. [42,64], who also observed airway inflammation and lower levels of inflammatory cells in the BALF after smoking cessation. It should be noted that it is very difficult to compare the numerous studies, since the smoking cessation period, the duration of smoking and the experimental set-up varied between the studies, which could lead to discrepancies. Additionally, several studies in COPD patients found a normalized cell count in the BALF and sputum after smoking cessation [24,25]. In contrast, other studies indicate that there is an ongoing airway inflammation in COPD patients who had stopped smoking [29-32]. These findings indicate that inflammatory changes in the airways of smoke-exposed mice are at least partially reversed after smoking cessation. The persistent airway inflammation (especially macrophages and lymphocytes) could be related to the irreversible tissue damage in the lungs, or to an ongoing microbial stimulus in the "sensitive" airways of smokers [65-67] as discussed by Willemse et al. [31]. Another explanation could be that COPD may have an autoimmune component that regulates the sustained airway inflammation after smoking cessation [68,69].

Little is known about cytokine and chemokine levels in the BALF after smoking cessation. To the best of our knowledge, this is the first reported *in vivo *study in which cytokine profiles were determined after cessation of cigarette smoke exposure. Increased levels of the pro-inflammatory cytokines IL-1α, IL-12 and TNF-α were observed in the BALF of cigarette smoke-exposed mice. IL-1α and TNF-α levels returned to basal levels after smoking cessation, while IL-12 was not normalized. The cytokines IL-1α, IL-12 and TNF-α are mainly produced by macrophages [70]. The alterations in these cytokine levels are in line with the accumulated macrophage levels before and reduced levels after smoking cessation. As IL-12 is a potent Th1 skewing cytokine, we suggest a Th1 polarization after cigarette smoke exposure. The decreased IL-10 levels after smoke exposure will amplify this polarization towards Th1, since IL-10 down-regulates the expression of Th1 cytokines [71]. Other authors also describe a possible association between COPD and a Th1-driven immune response [72,73]. Moreover, after smoking cessation the IL-10 levels were still significantly reduced compared to the air-exposed animals. IL-10 could also play a role in function and differentiation of the regulatory T cell, which is likely to be associated with the control of immune responses in COPD [74,75]. A significant increase of the CXCL2 concentration was observed in the lung homogenates of the smoke-exposed mice compared to the control animals. The CXCL2 increase is most probably important for the neutrophil recruitment to the lungs following cigarette smoke exposure, which is also indicated by Thatcher et al. [63]. The chemokines CCL2 and CCL3 were also elevated during COPD progression. This is in accordance with the accumulated macrophage, neutrophil and lymphocyte levels in the BALF of the smoke-exposed mice, since CCL2 is a monocyte chemoattractant and is produced by multiple cell types, including monocytes, macrophages, endothelial cells and epithelial cells [76]. CCL3 is mainly released by monocytes/macrophages and is involved in the recruitment and activation of pro-inflammatory cells, such as T-cells, monocytes/macrophages and neutrophils [77,78]. Like IL-12, the synthesis of CCL3 is typically associated with a Th1 milieu [79]. The CCL3 receptor, CCR1 is upregulated on Th1 cells by IL-12 [80,81], while CCR5, is primarily expressed on Th1 cells and promotes Th1 skewing [82,83]. Th1 cells secrete IL-2, IFN-у and TNF-α, which activate CD8+ T-cells. Since CCL3 attracts CD8+ lymphocytes, the elevated CCL3 in the smoke-exposed mice could be related to the increase in CD8+ T-cells seen in tissues of COPD patients [84]. These Th1-related cytokines and chemokines were markedly reduced after smoking cessation, suggesting that the Th1 skewing will diminish after smoking cessation.

Despite of the decrease in cell numbers and the reduction in cytokine and chemokine levels in the BALF after smoking cessation, the current study demonstrated that smoking cessation does not result in a profound reduction of airway inflammation, which is associated with the sustained emphysema. First, the neutrophils in the BALF were strongly reduced after smoking cessation to almost basal levels, but were still significantly increased compared to the control group. The macrophages in the alveolar cavity were also not completely restored toward basal levels after smoking cessation. Furthermore, the cigarette smoke-induced increase of lymphocytes was not changed after cessation of cigarette smoke exposure. Finally, the histological lung sections showed that the inflammatory cells and the brown-pigmented macrophages were still present in the lung tissue after smoking cessation of 8 weeks, confirming the results described by Seagrave et al. [64]. The pigmented macrophage has been a consistently reported inflammatory cell type in COPD and contains characteristic brown-pigmented cytoplasmic inclusions believed to be by-products of cigarette smoke [85-87]. It could be that these brown-pigmented macrophages together with the elevated lymphocytes in the BALF are responsible for the sustained airway inflammation observed in the lung tissue after smoking cessation. Future research is needed to investigate whether this ongoing inflammation is permanent after smoking cessation.

In conclusion, cigarette smoke exposure leads to irreversible lung damage and heart hypertrophy. The inflammatory changes in the airways caused by cigarette smoke exposure were only partially reversed after smoking cessation. Although smoking cessation should be the first step in reducing the progression of lung emphysema, additional medication could be provided to tackle the sustained airway inflammation.

## Competing interests

The authors declare that they have no competing interests.

## Authors' contributions

SB performed the experimental studies and was involved in acquisition and interpretation of data and drafted the manuscript. FP-N helped on the draft of the manuscript. PAJ-H, AD-K and GF supervised the study and contributed to the writing of the final paper. All authors read and approved the final manuscript.
